# Osteosclerotic changes on computed tomography predict disease progression and poor survival in prostate cancer with osteoblastic metastases

**DOI:** 10.1097/JS9.0000000000002758

**Published:** 2025-06-23

**Authors:** Meiting Chen, Zhenhua Yang, Shuai Yang, Jun Wang, YuPing Xie, Riqing Huang, Haifeng Li, Yonghong Li, Yanxia Shi, Sheng Li

**Affiliations:** a Department of Medical Oncology, State Key Laboratory of Oncology in South China, Guangdong Provincial Clinical Research Center for Cancer, Sun Yat-sen University Cancer Center, Guangzhou, China; b Department of Medical Oncology, Guangdong Provincial Hospital of Chinese Medicine, Zhuhai, China; c Jiangmen Wuyi Hospital of Traditional Chinese Medicine, Jiangmen, China; d Department of Medical Oncology, Guangdong Provincial Hospital of Chinese Medicine, Zhuhai, China; e Department of Urology, State Key Laboratory of Oncology in South China, Guangdong Provincial Clinical Research Center for Cancer, Sun Yat-sen University Cancer Center, Guangzhou, China; f Department of Radiology, State Key Laboratory of Oncology in South China, Guangdong Provincial Clinical Research Center for Cancer, Sun Yat-sen University Cancer Center, Guangzhou, China

**Keywords:** bone metastasis, computed tomography, osteosclerotic change, prostate cancer, tumor progression

## Abstract

**Background:**

Radiographic progression-free survival (rPFS) in prostate cancer (PC) may not always accurately reflect tumor progression. However, the prognostic significance of osteosclerotic changes (OCs) in PC remains unclear.

**Methods:**

A training cohort of 152 PC patients with osteoblastic metastases was recruited from the Sun Yat-sen University Cancer Center, while a validation cohort of 41 patients was obtained from two additional hospitals. Patients were stratified into two groups based on computed tomography findings: those with OCs and those without OCs (no osteosclerotic change [NOC]). Clinical outcomes were subsequently analyzed.

**Results:**

In the training cohort, 103 and 49 patients were classified into the OC and NOC groups, respectively. A significantly higher proportion of radiographic progression was observed in the OC group than in the NOC group (72.8% vs. 51.0%, *P* = 0.0105). The OC group demonstrated significantly worse rPFS and overall survival (OS) than the NOC group (median rPFS: 11.6 months vs. 52.9 months, *P* < 0.0001, median OS: 30.7 months vs. 67.1 months, *P* < 0.0001). Multivariate analysis identified OC as an independent prognostic factor for poor rPFS and OS. The results were validated in an external cohort.

**Conclusion:**

OCs are associated with adverse survival outcomes and may serve as potential biomarkers for disease progression and reduced survival in PC patients with osteoblastic metastases.

## Introduction

Bone metastasis is a frequent and clinically significant complication in men with metastatic prostate cancer (PC). It not only induces pain, physical disability, and functional deterioration but also plays a substantial role in elevating mortality rates^[1]^. The extent of bone metastasis is strongly correlated with survival outcomes, emphasizing the critical need for its accurate assessment^[2]^. The routine evaluation of bone metastases typically employs imaging techniques such as bone scintigraphy, computed tomography (CT), magnetic resonance imaging (MRI), and positron emission tomography (PET)^[3,4]^. According to the guidelines outlined by the Prostate Cancer Clinical Trials Working Group 3 (PCWG3), bone scintigraphy utilizing ^99m^Tc-methylene diphosphonate radionuclide is regarded as the standard imaging modality for detecting bone metastases^[5]^.

The current assessment methods for bone metastasis remain semi-quantitative and prone to variability owing to subjective interpretation. Radiographic progression is typically evaluated based on the emergence of new bone lesions or advancement of existing target lesions^[5]^. Some studies have proposed that radiographic progression-free survival (rPFS) or clinical progression-free survival could potentially serve as surrogate endpoints for overall survival (OS)^[6,7]^. However, it is important to note that the tumor flare phenomenon observed on bone scintigraphy can occasionally hinder the accurate assessment of tumor progression. Therefore, there is a pressing need for an innovative and cost-effective approach to evaluate the treatment response in the context of bone metastasis.

For lytic lesions, the assessment of bone response primarily adhered to the MD Anderson response criteria^[8,9]^. Extensive research has shown that osteosclerotic changes (OCs) in lytic bone lesions are associated with a favorable tumor response across various solid tumors, including lung and breast cancer^[10,11]^. Notably, patients with non-small cell lung cancer and lytic bone metastasis who exhibit early OCs tend to experience longer progression-free survival (PFS) than those without such changes^[11]^. A study involving 15 lung cancer patients further demonstrated that the early response of lytic bone metastasis was aligned with the overall tumor response in individuals treated with nivolumab^[12]^. Additionally, in patients with POEMS syndrome, increased sclerosis or the resolution of sclerotic lesions observed on CT scans serves as an indicator of a positive or negative tumor response, respectively^[13]^.

CT imaging is an indispensable diagnostic tool, offering critical information for the evaluation of lytic bone lesions. Nevertheless, the current literature reveals a significant gap in the assessment of osteoblastic metastasis using CT imaging, particularly in the context of PC. To address this limitation, our study specifically investigated PC patients with bone metastasis and conducted a comprehensive analysis of the correlation between OCs and patient survival outcomes. This study aimed to develop a robust and prostate-cancer-specific framework for bone response assessment, thereby enhancing the clinical evaluation of metastatic progression in this patient population. This cohort study has been reported in line with the STROCSS criteria^[14]^.

## Materials and methods

### Study design

This study was conducted at the Sun Yat-sen University Cancer Center from July 2010 to May 2023, with a training cohort consisting of 176 patients diagnosed with prostate adenocarcinoma and confirmed bone metastasis. The institutional review board of Sun Yat-sen University Cancer Center approved the study protocol. This cohort study has been reported in line with the STROCSS criteria^[14]^. The inclusion criteria were histopathological confirmation of prostate adenocarcinoma and radiologically verified bone metastasis, assessed through bone scintigraphy and CT imaging from the initiation of systemic therapy until disease progression. Bone metastasis was diagnosed through histopathological examination of biopsy specimens or characteristic imaging findings suggestive of metastatic disease, particularly in cases of multiple osseous lesions and typical radiographic features in patients with confirmed primary prostate cancer. In our clinical practice, the diagnostic confirmation of osseous metastasis primarily relies on bone scintigraphy combined with CT imaging, with some cases additionally evaluated using prostate-specific membrane antigen (PSMA)-PET/CT. Radiographic indicators of metastatic bone disease included: (1) permeative or non-geographic bone destruction patterns; (2) wide transitional zones between normal and affected bone; (3) absent or interrupted sclerotic margins; (4) aggressive periosteal reactions; and (5) cortical disruption with associated soft tissue masses. Osteoblastic metastases typically present as sclerotic lesions, occasionally accompanied by visible fracture lines and disruption of the normal anatomical alignment.

**HIGHLIGHTS**

- Osteosclerotic changes (OCs) on computed tomography (CT) scans are predictive of disease progression and poor survival in prostate cancer patients with osteoblastic metastases.
- Patients with OCs exhibited significantly shorter radiographic progression-free survival and overall survival compared to those without OCs.
- OCs were identified as an independent prognostic factor for adverse outcomes in both training and validation cohorts.
- CT-based assessment of OCs offers a practical and cost-effective alternative to traditional bone scintigraphy for evaluating bone metastasis progression.
- This study underscores the potential of OCs as early biomarkers for disease progression in prostate cancer, highlighting the need for further prospective validation.

Patients were eligible to receive radiotherapy or surgical intervention for bone metastasis provided that untreated metastatic lesions were present. The administration of bone-modifying agents, including bisphosphonates or denosumab, was also permitted. The exclusion criteria were as follows: (1) a documented history of any other primary malignancy, (2) incomplete CT imaging data or clinical documentation, and (3) prior radiation therapy or surgical treatment of the target bone lesions. To ensure the robustness of our findings, we established an independent validation cohort that was retrospectively recruited from two medical institutions: the Cancer Center of Fifth Hospital of Sun Yat-sen University and Jiangmen Wuyi Hospital of Traditional Chinese Medicine. Comprehensive data collection included demographic information, tumor characteristics, therapeutic interventions, routine laboratory parameters, serum tumor marker profiles, bone scintigraphy findings, whole-body CT imaging data, and detailed treatment protocols.

Therapeutic strategies were determined by experienced oncologists in accordance with established prostate cancer management guidelines^[15,16]^. Standard endocrine therapy regimens consist of next-generation androgen receptor signaling inhibitors (abiraterone, enzalutamide, apalutamide, or darolutamide) in combination with androgen deprivation therapy (ADT). The predominant treatment approach for patients progressing to castration-resistant prostate cancer (CRPC) involves docetaxel-based chemotherapy regimens.

### Outcome and follow-up

Radiographic assessments were conducted using CT and bone scintigraphy at the initiation of systemic therapy and subsequently every 12 weeks until disease progression or the onset of intolerable toxicity. Each patient underwent non-enhanced and contrast-enhanced CT of the chest, abdomen, and pelvis. CT images were acquired using various systems, including a 64-slice spiral CT system (Aquilion TSX-101A, Toshiba Medical System, Otawara, Japan), 128-slice spiral CT system (Discovery CT750 HD, GE System, Milwaukee, WI, USA), 256-slice spiral CT system (Brilliance iCT, Philips System, Cleveland, OH, USA), or dual-source spiral CT system (SOMATOM Force, Siemens Medical System, Erlangen, Germany). Contrast-enhanced CT scans were performed subsequent to an intravenous bolus injection (300 mg–450 mg/kg) of a nonionic iodinated contrast agent administered into the antecubital vein at a rate of 3.0 ml/s via a high-pressure syringe. The details for CT parameter are illuminated in Supplementary materials digital content available at: http://links.lww.com/JS9/E517.

The primary endpoint of our study was rPFS, which was rigorously assessed in accordance with the PCWG3 guidelines and Response Evaluation Criteria in Solid Tumors (RECIST) version 1.1. rPFS was calculated from the initiation of systemic therapy until either radiological disease progression or death from any cause, whichever event occurred first. For patients with measurable lesions, tumor response was assessed according to RECIST 1.1. Secondary endpoints included OS and prostate-specific antigen (PSA) response rate. OS was calculated from treatment initiation to death or the last follow-up. Patients were censored at their last tumor assessment if they remained progression-free or initiated subsequent therapy without documented progression. Death without prior progression was defined as progression at the time of death. PSA response was defined as a ≥50% reduction from baseline levels. Bone metastasis burden was classified using the Extent of Disease (EOD) score based on bone scintigraphy as follows: 0 (none), 1 (1–5 lesions), 2 (6–20 lesions), 3 (>20 lesions, excluding super scan), and 4 (super scan).

### Assessment of the bone response

**Figure 1.**
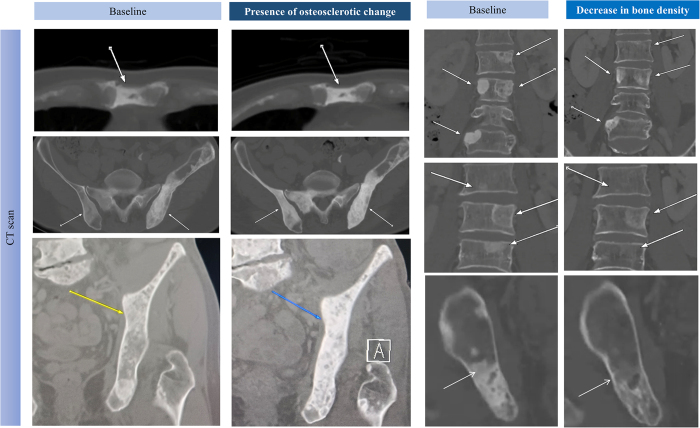
Typical image on assessment of osteosclerotic changes (OCs), and no osteosclerotic changes (NOC) in prostate cancer with osteoblastic metastasis. CT attenuation of the lesion increased over 10% in bone lesions defined as OC, otherwise NOC. Besides, in three cases with OCs, the radiography progression-free survival (rPFS) was 1.4, 14.0, and 9.1, respectively. In three cases with NOCs, the rPFS was 26.5, 10.0, and 30.4, respectively.

Two radiologists with 16 and 10 years of experience independently reviewed the pre- and post-systemic therapy CT scans for each patient. Both readers were blinded to each other’s findings and to the treatment details, including the regimen, number of cycles, and therapeutic outcomes. The evaluation focused on two primary aspects: assessment of bone lesions and efficacy of systemic therapy. For any inconsistencies, they reread the CT images together, inviting a third radiologist if necessary, to reach a consensus. All images were analyzed using standard bone window settings (window level: 200 Hounsfield units [HU]; window width: 2000 HU) on non-contrast CT scans. A qualitative evaluation of each lesion on the pre-systemic therapy CT scan was conducted to classify them as lytic, osteoblastic, or mixed, based on their appearance relative to the adjacent normal bone^[17]^. OCs were first checked for all existing typical metastatic bone lesions using visual assessment. For potential OCs observed visually, measurements of each individual lesion were recorded and analyzed. To quantify the OCs, a region of interest (ROI) was delineated to encompass the entire lesion on the axial images, allowing for the measurement of the average CT attenuation in HU. Radiologists identified OCs by measuring the increase in the average CT attenuation within the ROI in bone lesions from the initial scan. The same ROI location was consistently used in the follow-up studies for each patient. Based on prior research, a cut-off value of 10% was applied to differentiate between the OC group and no osteosclerotic change (NOC) group^[11]^. CT scans of bone metastases before and after systemic therapy were compared to evaluate the changes in CT attenuation. An increase in the average CT attenuation within the ROI of more than 10% was classified as the OC group, whereas an increase of less than 10% was categorized as the no NOC group. For patients with multiple osseous metastatic lesions, classification into the OC group was based on at least one lesion demonstrating an increase in HU of more than 10%. Conversely, if all lesions showed an increase in HU of less than 10%, the patient was assigned to the NOC group. In this study, OC assessment was limited to pre-existing lesions; new lesions identified on follow-up CT scans were recorded, but not evaluated for OCs. Representative CT images illustrating these classifications are shown in Figure 1. The details for CT parameter and OC measurement are illuminated in Supplementary materials digital content available at: http://links.lww.com/JS9/E517.

### Statistical analysis

The study population included patients who were enrolled and had complete CT imaging and clinical data profiles. Descriptive statistics were used to summarize patient characteristics, treatment details, and anti-tumor activity. Radiographic progression was compared between the OC and NOC groups using the chi-squared (χ^2^) test. Survival analyses for rPFS and OS were performed using the Kaplan–Meier method. Inter-reader reliability was quantified using Cohen’s kappa for categorical variables. Univariate Cox regression analyses and multivariate proportional hazards regression models were used to identify independent prognostic factors. All reported *P-*values were two-sided, with a significance threshold set at *P* < 0.05.

## Results

### Baseline characteristics

**Figure 2.**
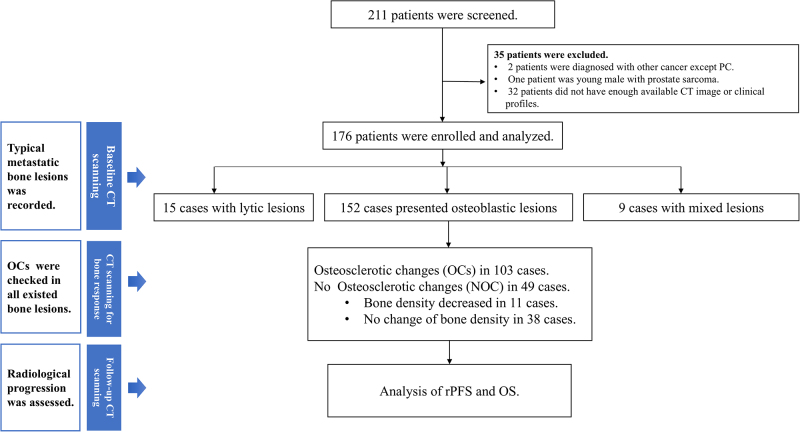
Flow diagram.

The study design is illustrated in Figure 2. After screening 211 patients, 176 were ultimately included and analyzed. Based on the initial presentation of bone lesions, 152 patients exhibited osteoblastic lesions, while 15 and 9 patients presented with lytic lesions and mixed lesions, respectively. Table 1 summarizes the baseline characteristics of all enrolled patients in the training cohort. The specific systemic therapy regimens for all the patients are shown in Supplemental Digital Content Table S1, available at: http://links.lww.com/JS9/E451. During the bone response assessment, 54.0% of the patients received novel hormone therapy, including agents such as abiraterone, enzalutamide, apalutamide, rezvilutamide, proxalutamide, and darolutamide, while 19.9% received docetaxel-based chemotherapy. The bone response analysis primarily focused on 152 patients presenting with osteoblastic lesions. Among these patients, OCs were observed in 67.8% of the cases, with all OC lesions identified via visual assessment showing an increase in HU exceeding 10%. Inter-rater reliability between the two radiologists showed substantial agreement (Cohen’s *κ* = 0.69, 95% confidence interval [CI]: 0.57–0.81, *P* < 0.001). As of the data cut-off date of December 1, 2024, the median follow-up duration is 53.8 months (95% CI: 42.2–70.3 months).

**Table 1:** Baseline characteristics of the training cohort

	Osteoblastic (*N* = 152)	Lytic (*N* = 15)	Mixed (*N* = 9)	Overall (*N* = 176)
Age				
Mean (SD)	67.9 (8.01)	69.3 (7.21)	67.2 (9.78)	68.0 (8.00)
Median [min, max]	68.0 [48.0, 85.0]	69.0 [55.0, 88.0]	69.0 [51.0, 81.0]	68.0 [48.0, 88.0]
Osteosclerotic change	103 (67.8%)	12 (80.0%)	4 (44.4%)	107 (60.8%)
No osteosclerotic change				
Bone density decreased	11 (7.2%)	1 (6.7%)	2 (22.2%)	23 (13.1%)
Unchanged	38 (25.0%)	2 (13.3%)	3 (33.3%)	46 (26.1%)
ECOG				
0–1	146 (96.1%)	15 (100%)	9 (100%)	170 (96.6%)
≥2	6 (3.9%)	0 (0%)	0 (0%)	6 (3.4%)
EOD scores				
1	29 (19.1%)	9 (60.0%)	1 (11.1%)	39 (22.2%)
2	92 (60.5%)	6 (40.0%)	8 (88.9%)	106 (60.2%)
3	30 (19.7%)	0 (0%)	0 (0%)	30 (17.0%)
4	1 (0.7%)	0 (0%)	0 (0%)	1 (0.6%)
Metastasis				
Bone metastasis only	83(54.6%)	6(40.0%)	2(22.2%)	91(51.7%)
Visceral	31 (20.4%)	2 (13.3%)	4 (44.4%)	37 (21.0%)
Pelvic nodes	69 (45.4%)	7 (46.7%)	6 (66.7%)	82 (46.6%)
Extrapelvic nodes	50 (32.9%)	4 (26.7%)	3 (33.3%)	57 (32.4%)
Liver	9 (5.9%)	1 (6.7%)	2 (22.2%)	12 (6.8%)
Lung	23 (15.1%)	1 (6.7%)	3 (33.3%)	27 (15.3%)
Brain	2 (1.3%)	0 (0%)	0 (0%)	2 (1.1%)
PSA level at study entry (ng/mL) median [min, max]	68.7 [0.0550, 5000]	287 [2.08, 5000]	94.7 [3.72, 4950]	84.7 [0.0550, 5000]
Denosumab or zoledronic acid use	110 (72.4%)	14 (93.3%)	8 (88.9%)	132 (75.0%)
Regional therapy on bone				
Surgery	13 (8.6%)	2 (13.3%)	1 (11.1%)	16 (9.1%)
Radiotherapy	62 (40.8%)	8 (53.3%)	2 (22.2%)	72 (40.9%)
Systemic therapy				
Endocrine therapy	111 (73.0%)	13 (86.7%)	8 (88.9%)	132 (75.0%)
Novel hormone therapy	80 (52.6%)	8 (53.3%)	7 (77.8%)	95 (54.0%)
Docetaxel-based chemotherapy	33 (21.7%)	1 (6.7%)	1 (11.1%)	35 (19.9%)

Novel hormone therapies included abiraterone, enzalutamide, apalutamide, rezvilutamide, proxalutamide, and darolutamide.

ECOG, Eastern Cooperative Oncology Group; EOD, extent of disease; PSA, prostate-specific antigen.

### Correlation between the OC, progression, and survival outcomes

**Figure 3.**
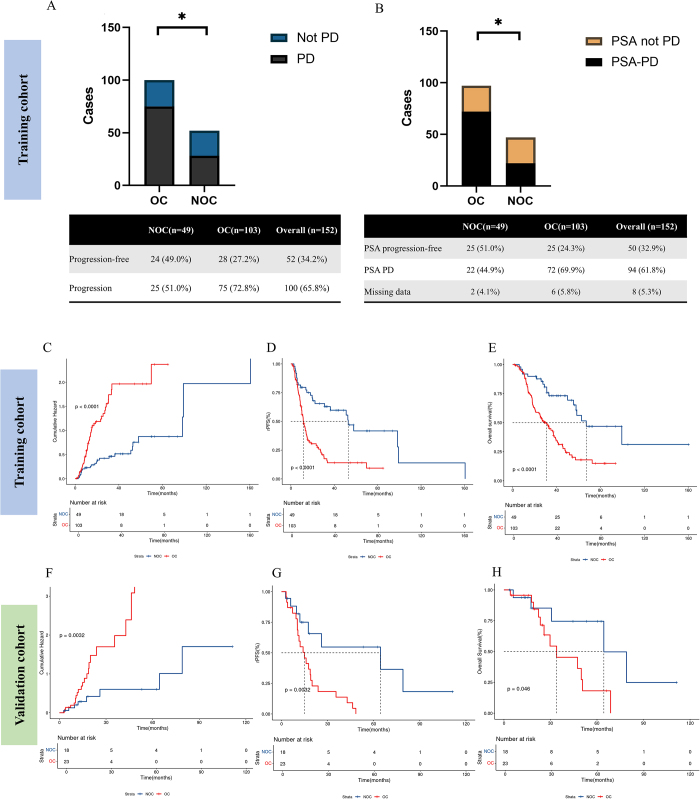
Proportion of radiography progression (A) and PSA progression (B) in patients with osteosclerotic change (OC) and no osteosclerotic change (NOC). Cumulative hazard (C), radiography progression-free survival (rPFS) (D), and overall survival (OS) (E) of patients with osteoblastic metastasis in the training cohort in the OC and NOC group. Cumulative hazard (E), rPFS (F), and OS (G) for the OC and NOC group in patients with osteoblastic metastasis in the validation cohort.

The analysis revealed a significantly higher proportion of radiographic progression in patients with OCs than in those without OCs (NOC) (72.8% vs. 51.0%, *P* = 0.0105; Fig. 3A). Similarly, a significantly higher proportion of PSA progression (PSA PD) was observed in the OC group than that in the NOC group (69.9%, 95% CI: 60.0%–78.5% vs. 44.9%, 95% CI: 30.7%–59.8%; *P* = 0.0016; Fig. 3B), indicating that the trend of OC was aligned with PSA progression. Additionally, a significantly lower proportion of PSA responses was observed in patients with OC than in those with NOC (54.4%, 95% CI: 44.2%–64.2% vs. 81.6%, 95% CI: 68.0%–91.2%; *P* = 0.002; Supplemental Digital Content Figure S1, available at: http://links.lww.com/JS9/E450 and Supplemental Digital Content Table S2 available at: http://links.lww.com/JS9/E451).

**Figure 4.**
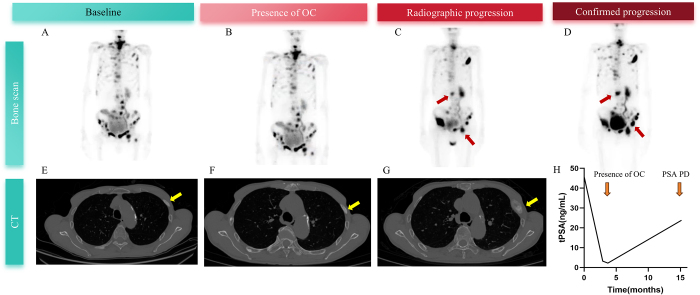
Bone scans (A–C) and CT scans of the rib (E–G) of Patient #120 at baseline, during treatment with enzalutamide, and radiographic progression. Radiographic progression was confirmed after 9 weeks by ECT scanning (D). The evaluation of the serum total PSA concentration is shown in H.

The cumulative hazard ratio (HR), rPFS, and rPFS based on OCs in the training cohort are illustrated in Figure 3C–E. Among all rPFS events in the study, 20 cases presented with new lesions, 56 cases showed bone scintigraphy progression, 23 cases exhibited target lesion enlargement, and one case demonstrated both new lesions and target lesion enlargement. The median rPFS for patients with OCs is 11.6 months, compared to 52.9 months for those without OCs (HR = 2.23, 95% CI: 1.76–3.87, *P* < 0.0001). Similarly, the median OS for patients with OCs is 30.7 months, versus 67.1 months for those without OCs (HR = 2.85, 95% CI: 1.87–4.34; *P* < 0.0001). A representative case is shown in Figure 4. Patient #120 developed OCs 3.7 months after initiating treatment with enzalutamide, 11.5 months prior to bone scintigraphy progression, accompanied by an increase in PSA levels (Fig. 4).

In the lytic group, OCs were observed in 12 of 15 patients. Among the mixed group, which consisted of patients with a comparable proportion of lytic and osteoblastic lesions, four patients exhibited OCs following systemic therapy. However, due to the small sample size of only nine patients in the mixed group, survival analysis was not conducted for this subgroup. Analyses of OC across the different subgroups are presented in Supplemental Digital Content Figure S1, available at: http://links.lww.com/JS9/E450and Supplemental Digital Content Tables S2 and S3 available at: http://links.lww.com/JS9/E451.

### Efficacy of OC in predicting prognosis

**Figure 5.**
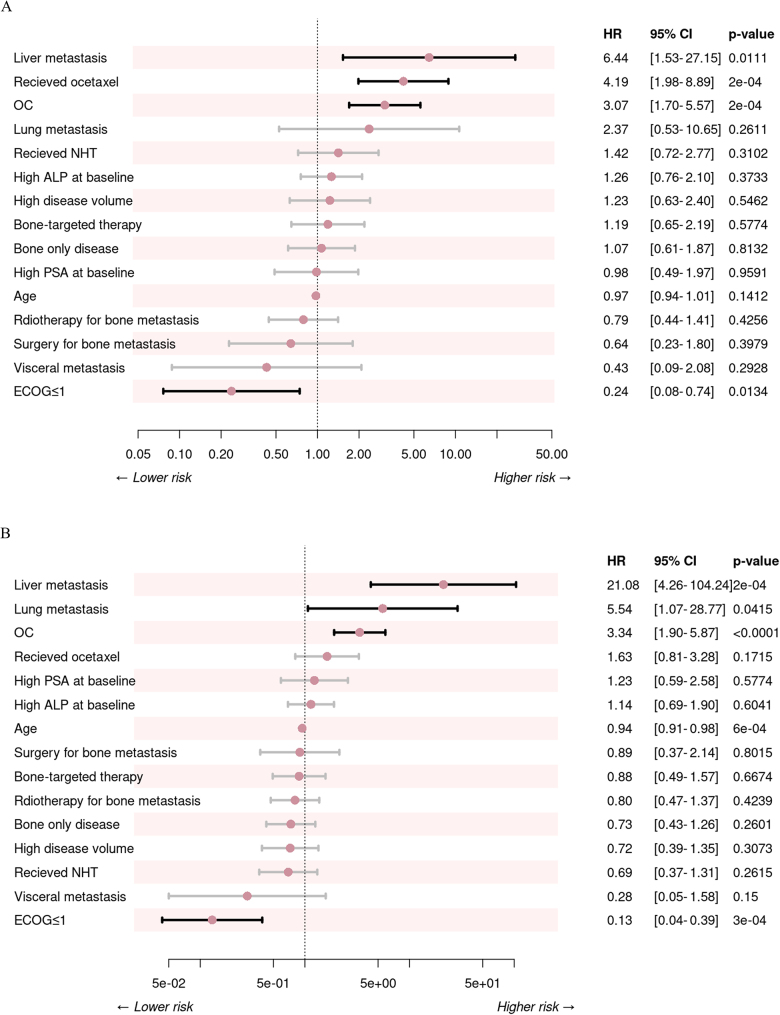
The forest plots for multivariate analysis on OS (A) and rPFS (B).

Univariate analysis identified several clinical factors significantly associated with poor survival, including the presence of OCs, docetaxel chemotherapy, liver metastasis, and bone-only metastasis. Conversely, an Eastern Cooperative Oncology Group (ECOG) performance status score of ≤1 was associated with improved OS. In a subsequent forward conditional Cox regression analysis, four variables retained their negative prognostic impact on OS: presence of OCs, receiving docetaxel chemotherapy, presence of liver metastasis, and an ECOG performance status score of ≥1 (Fig. 5A). Additional details of these prognostic factors are provided in Supplemental Digital Content Table S4, available at: http://links.lww.com/JS9/E451.

Univariate analysis revealed that OC, younger age, presence of liver metastasis, and receiving docetaxel chemotherapy were associated with inferior rPFS. Multivariate analysis further confirmed that OCs, age, liver metastasis, lung metastasis, and ECOG performance status score were significantly associated with inferior rPFS (Fig. 5B). Detailed results are provided in Supplemental Digital Content Table S5, available at: http://links.lww.com/JS9/E451. Two OC-based nomograms demonstrating their predictive performance for rPFS and OS are presented in Supplemental Digital Content Figure S2A and B, available at: http://links.lww.com/JS9/E450, respectively. The sensitivity analysis excluding docetaxel-treated patients (*n* = 92) showed that the prognostic association of OC parameters remained significant, and maintained discriminative ability (concordance = 0.73) (Supplemental Digital Content Table S7, available at: http://links.lww.com/JS9/E451).

### External validation and survival prediction

To validate our findings, we included 43 patients from two additional hospitals. The characteristics of the patients are presented in Supplemental Digital Content Table S6, available at: http://links.lww.com/JS9/E451. A high degree of consistency was observed between validation and training cohorts. In the validation cohort, we analyzed 41 patients with osteoblastic metastases. OCs were observed in 56.1% of the patients with osteoblastic lesions. The analysis of cumulative hazard, rPFS, and OS based on OCs in patients with osteoblastic lesions is shown in Figure 3F–H. Specifically, the rPFS for patients with and without OCs was 14.9 months and 64.4 months, respectively (HR = 3.11, 95% CI 1.46–6.62; *P* = 0.0032). Similarly, the median OS for patients with OC and NOC was 34.0 months and 64.4 months, respectively (HR = 2.78, 95% CI 1.02–7.58; *P* = 0.046). These results suggested the potential predictive value of OCs in this cohort.

## Discussion

In our study, we analyzed 176 patients with advanced prostate cancer to assess bone response using CT scans. Our findings suggested that the presence of OCs within osteoblastic lesions may serve as potential indicators of disease progression. The observed associations between OCs and poorer rPFS and OS highlight their possible utility as early biomarkers for adverse outcomes. Multivariate analysis indicated that OCs were independently associated with survival outcomes and disease progression. The predictive value of OCs was further validated in an external cohort. Therefore, our findings suggest that CT-based OC assessment could address a critical gap in current bone metastasis evaluation, particularly in resource-limited settings where advanced imaging (e.g. PSMA-PET) may be unavailable, though further validation is warranted.

Bone metastasis occurs in approximately 90% of patients with advanced prostate cancer, and is a leading cause of mortality in this population^[18,19]^. Recent studies have highlighted the potential of next-generation imaging techniques, such as PET/CT with prostate cancer-specific tracers such as^[18]^ F-fluciclovine, for the early and sensitive detection of bone metastases^[20]^. However, challenges related to accessibility, cost, and unclear follow-up response assessments with PET/CT or MRI persist. Currently, bone scintigraphy remains the standard for evaluating bone lesions according to the PCWG3 criteria despite its limitations in objectivity and quantification^[21]^. Emerging techniques, such as the automated bone scan index (aBSI), have shown promise in quantifying bone metastases and correlating them with prognosis in metastatic CRPC^[22]^. Retrospective studies have suggested that an increase in aBSI may be associated with inferior OS^[23]^. While aBSI has demonstrated predictive value for the time to CRPC in metastatic hormone-sensitive prostate cancer, its utility is limited by the time required to confirm disease progression due to bone scintigraphy flares and its inability to assess visceral and lymph node metastases^[24]^. Additionally, the analytical validation of aBSI using EXINI boneBSI version 2.0 is costly and unavailable in China, hindering its widespread application and raising uncertainties about its predictive value in Chinese and Asian populations^[25]^. Notably, aBSI’s predictive performance of aBSI for OS appears to vary by ethnicity, with poorer outcomes observed in Asian populations than in Caucasian populations^[25]^. The EOD score has shown utility in identifying high-risk patients with de novo metastatic prostate cancer; however, its role in therapeutic response evaluation remains controversial^[26]^. Consequently, further research is needed to clarify the utility of aBSI and EOD in assessing treatment responses. We propose that OCs provide a practical alternative to bone scintigraphy, addressing limitations of current imaging modalities. OC assessment holds particular clinical utility in scenarios where conventional response metrics are ambiguous, such as during long-term ADT or androgen receptor pathway inhibitor treatment, where patients often lack measurable soft-tissue lesions and PSA levels may remain low or fluctuate obscurely. In these cases, serial OC evaluation provides an accessible and objective biomarker to monitor anti-tumor activity, complementing PSA and standard imaging. Similarly, in CRPC patients with worsening symptoms without meeting radiographic progression criteria, OC dynamics may help discern true disease activity and inform earlier therapeutic adjustments. To align with PCWG3 criteria, OCs could serve as a complementary biomarker. In the absence of new lesions or tumor enlargement, OC progression may help evaluate treatment response, especially in cases with discordant PSA levels, where it could clarify disease status alongside clinical symptoms. Additionally, OC assessment offers a viable option when bone scintigraphy or PSMA PET-CT is unavailable. Incorporating OCs into response criteria could improve progression assessment accuracy, particularly when traditional metrics are inconclusive.

Serum PSA levels are commonly used to predict tumor response and disease progression; however, approximately 20.5% of patients experience a PSA flare, complicating interpretation^[27]^. The evaluation and management of metastatic disease relies on a comprehensive approach that incorporates serum PSA levels, radiographic findings, and clinical symptoms. Consequently, there is an ongoing exploration of reliable surrogate markers to assess treatment response. OCs, characterized by increased bone density or dense sclerosis on CT scans, have been identified as markers of favorable response and improved survival in patients with lung cancer osteolytic metastases^[28]^. In contrast to bone metastases from other cancers, prostate cancer bone metastases are predominantly osteoblastic rather than osteolytic, driven by the unique mechanisms of bone remodeling and abnormal new bone formation^[29–31]^. This distinction suggests differences in the bone response to OCs in prostate cancer compared with other malignancies. Our study demonstrated that OCs within osteoblastic lesions are associated with PSA PD, worse OS, and likely indicate true disease progression. The underlying mechanisms responsible for the differing behaviors of OCs in prostate cancer versus lung cancer warrant further investigation. Additionally, factors such as age, ECOG performance status, and liver metastasis were associated with poor prognosis, consistent with recent studies^[32–34]^. Patients with liver metastasis exhibit a worse prognosis than those with metastases at other sites, potentially due to more aggressive genomic features^[35]^. OCs may serve as an early indicator of disease progression in patients with concurrent liver and bone metastases, although prospective studies are needed to validate this hypothesis.

Several studies suggested that OCs are associated with favorable treatment response in lytic metastasis from lung or breast cancer^[10–12,28]^. However, our study reveals a paradoxical association in prostate cancer, where OCs in osteoblastic lesions correlate with disease progression and poorer outcomes. This divergence likely comes from fundamental differences in the bone metastatic microenvironment^[36,37]^. Osteoclasts play crucial roles in osteolytic bone metastasis, by digesting bone matrix and indirectly enhancing tumor colonization in lung or breast cancer^[36,37]^. In contrast, prostate cancer bone metastases are intrinsically osteoblastic, driven by tumor-secreted factors that dysregulate bone remodeling^[38]^. Therefore, we suggested that the observed OCs in our cohort may represent pathological osteosclerosis as consequence of tumor-induced osteoblast hyperactivity that further disrupts bone homeostasis, which contributes to treatment resistance. A deeper understanding of the mechanism for osteosclerosis and prostate cancer cells would be helpful in the future.

This study had several limitations that warrant consideration. First, its retrospective design introduces the potential for bias, compounded by the heterogeneity in baseline characteristics and treatment factors among the study population. The variability in the systemic treatments administered to patients further exacerbates this issue. Although confounding factors including liver metastasis and docetaxel therapy could affect interpretation, sensitivity analyses consistently showed the reliability of OC measurements independent of docetaxel treatment. While rigorous quality control measures were implemented, including daily scanner calibration and annual uniformity testing, the inclusion of different CT scanners may introduce calibration bias and technical variability. In our study, OC analysis focused on the change in HU values between serial scans for each individual patient, with all longitudinal comparisons performed using the same scanner model, thereby minimizing inter-scanner variability when assessing treatment response. The assumption that patients without follow-up CT scans did not exhibit a bone response could have led to an underestimation of the bone response rates. Furthermore, inconsistencies in the timing of CT scans due to variations in measurement protocols may have introduced heterogeneity. While our methodology aimed to assess the clinical significance of OC differences and their impact on long-term survival outcomes, the study remains susceptible to residual confounding from unmeasured variables, which could bias the effect estimates. Besides, the modest sample size of our validation cohort may still affect the statistical power of our conclusions, although we observed consistent trends between training and validation sets. Larger-scale validation studies are needed to confirm these findings and prospective studies are currently underway to address this need. These limitations warrant cautious interpretation of the results while highlighting important directions for future research.

Our study identified OCs on CT as surrogates for bone response and disease progression, particularly in patients unable to undergo bone scintigraphy. OCs offer a simple, reliable assessment for radiologists, with a >10% HU increase in bone windows that are easily detectable, enabling widespread clinical application. Future multicenter trials should validate the 10% HU threshold and explore automated quantification tools to standardize OC assessment. By incorporating OCs into PCWG3 criteria as a complementary biomarker in the future, it could be helpful when current assessment is ambiguities, particularly in cases with discordant PSA or scintigraphy results.

## Conclusion

Our study highlights the clinical significance of OC on CT in prostate cancer patients with osteoblastic metastases, as these changes are associated with inferior rPFS and OS and serve as early biomarkers of disease progression. Future prospective studies are essential to further evaluate the role of CT-based bone-response assessment and its broader clinical implications in prostate cancer management.

## Data Availability

The corresponding author had full access to all the data in the study and takes responsibility for the integrity of the data and accuracy of the data analysis. The datasets generated in the current study are available from the corresponding author upon reasonable request.

## Research registration unique identifying number (UIN)

The study is registered at ClinicalTrials.gov. (NCT06883474).

## Protocols

The details protocols for CT parameters and OC measurement are illuminated in Supplementary materials.
